# Fatty Pancreas and Risk of Type 2 Diabetes, Chronic Kidney Disease and Cardiovascular Events: Evidence From a Population‐Based Cohort

**DOI:** 10.1002/ueg2.70253

**Published:** 2026-07-02

**Authors:** Nicola Pugliese, Oveis Jamialahmadi, Arturo Cesaro, Valentina Flagiello, Rosellina M. Mancina, Alessio Aghemo, Stefano Romeo

**Affiliations:** ^1^ Department of Medicine (H7), Centre for Reproduction, Metabolism and Molecular Medicine (CeRM) Karolinska Institutet Huddinge Sweden; ^2^ Division of Internal Medicine and Hepatology Department of Gastroenterology IRCCS Humanitas Research Hospital Rozzano Italy; ^3^ Department of Translational Medical Sciences University of Campania ‘Luigi Vanvitelli’ Naples Italy; ^4^ Division of Cardiology A.O.R.N. ‘Sant'Anna e San Sebastiano’ Caserta Italy; ^5^ Operative Research Unit of Clinical Medicine and Hepatology Fondazione Policlinico Universitario Campus Bio‐Medico Roma Italy; ^6^ Department of Medicine and Surgery Universita Campus Bio‐Medico Research Unit of Clinical Medicine and Hepatology Roma Italy; ^7^ Department of Molecular and Clinical Medicine University of Gothenburg Gothenburg Sweden; ^8^ Department of Life Science, Health, and Health Professions Link Campus University Rome Italy; ^9^ Department of Biomedical Sciences Humanitas University Pieve Emanuele Italy; ^10^ Department of Endocrinology Karolinska University Hospital Huddinge Sweden; ^11^ Department of Medical and Surgical Sciences Magna Graecia University Catanzaro Italy; ^12^ Department of Cardiology Sahlgrenska University Hospital Gothenburg Sweden

**Keywords:** beta cell dysfunction, cardiometabolic risk, ectopic fat, insulin resistance, metabolic risk stratification, pancreatic steatosis

## Abstract

**Background:**

Fatty pancreas is a metabolically active ectopic fat depot, but its cardiometabolic implications have been assessed using heterogeneous thresholds. We investigated the association of fatty pancreas, quantified using MRI‐derived proton density fat fraction (PDFF) and categorised according to 2026 international consensus thresholds, with prevalent and incident type 2 diabetes (T2D), chronic kidney disease (CKD) and major adverse cardiovascular events (MACE).

**Methods:**

We analysed 19,255 European‐ancestry participants from the UK Biobank imaging sub‐study. Pancreatic PDFF was categorised as normal (< 6%), mild (6 to < 16%) and moderate‐to‐severe fatty pancreas (≥ 16%). Outcomes were ascertained through national health records. Associations were estimated using multivariable logistic regression and Cox models, adjusted for age, sex, BMI‐defined obesity, elevated MRI‐derived visceral adipose tissue and outcome‐specific covariates.

**Results:**

Moderate‐to‐severe fatty pancreas was associated with prevalent and incident T2D (OR 3.25, 95% CI 2.49–4.27; *p* < 0.001; HR 2.72, 1.66–4.46; *p* < 0.001), incident CKD (HR 1.82, 1.29–2.57; *p* < 0.001), and prevalent and incident MACE (OR 1.26, 1.04–1.56; *p* = 0.022; HR 1.30, 1.02–1.66; *p* = 0.034). Mild fatty pancreas was associated with incident T2D (HR 2.19, 1.40–3.42; *p* < 0.001) and incident MACE (HR 1.29, 1.06–1.59; *p* = 0.013). Each 5% increase in pancreatic PDFF was associated with higher odds and hazard of T2D (OR 1.16, 1.12–1.21; HR 1.17, 1.09–1.25; both *p* < 0.001).

**Conclusions:**

Fatty pancreas was independently associated with prevalent and incident T2D, incident CKD and, more modestly, with MACE. These findings position fatty pancreas within the cardiovascular‐kidney‐metabolic continuum and support the clinical relevance of consensus‐based PDFF thresholds for cardiometabolic risk assessment in European‐ancestry populations.

## Key Summary

- Summarise the established knowledge on this subject
  ◦ Fatty pancreas has been implicated in beta cell dysfunction, impaired insulin secretion and insulin resistance.
  ◦ A 2026 international multi‐society consensus endorsed by United European Gastroenterology and other leading societies established MRI‐derived PDFF thresholds for diagnosing and categorising fatty pancreas and highlighted the need for validation in large prospective cohorts.
  ◦ Previous studies on fatty pancreas and cardiometabolic outcomes have relied on heterogeneous, nonstandardised diagnostic thresholds.
- What are the significant and/or new findings of this study?
  ◦ In 19,255 European‐ancestry UK Biobank participants, consensus‐defined fatty pancreas was independently associated with prevalent and incident type 2 diabetes in a graded pattern, with up to 3‐fold higher risk for moderate‐to‐severe fatty pancreas and an approximately 17% higher risk per 5% PDFF increment.
  ◦ Moderate‐to‐severe fatty pancreas was associated with incident chronic kidney disease, and more modestly with prevalent and incident major adverse cardiovascular events, supporting its role within the cardiovascular‐kidney‐metabolic continuum.
  ◦ Mild fatty pancreas was also independently associated with incident type 2 diabetes and incident major adverse cardiovascular events.

## Graphical abstract

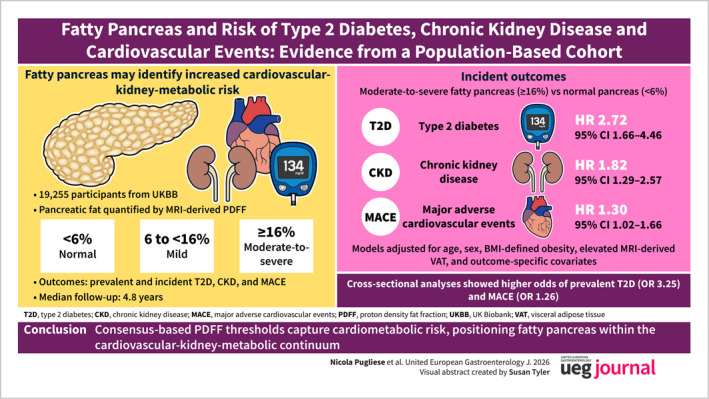

**Abbreviations**
CKD: chronic kidney disease
MACE: major adverse cardiovascular events
OPCS‐4: Office of Population Censuses and Surveys Classification of Interventions and Procedures, version 4
PDFF: proton density fat fraction
T2D: type 2 diabetes
UKBB: UK Biobank
VAT: visceral adipose tissue

## 1 Introduction

Fatty pancreas, defined as excessive fat accumulation within pancreatic tissue, is increasingly recognised as a manifestation of systemic metabolic dysfunction [1]. It affects approximately one in five adults and has been linked to cardiometabolic risk through lipotoxic, inflammatory, and endocrine mechanisms [1, 2, 3]. Intrapancreatic lipid accumulation may impair insulin secretion and beta‐cell function, while the associated systemic metabolic environment, characterised by dyslipidaemia, oxidative stress, chronic low‐grade inflammation, and endothelial dysfunction, may contribute to kidney and cardiovascular injury [4, 5, 6, 7].

Clinical evidence is consistent with these mechanisms. Prospective studies have linked fatty pancreas to the subsequent development of diabetes mellitus, and UK Biobank (UKBB)‐based analyses have reported graded associations between MRI‐derived intrapancreatic fat and diabetes mellitus, as well as with exocrine pancreatic diseases [8, 9, 10]. Evidence linking pancreatic fat to chronic kidney disease (CKD) and cardiovascular outcomes is also emerging, although data remain limited and heterogeneous [11, 12, 13]. However, prior studies have generally relied on empirically derived or study‐specific diagnostic thresholds rather than standardised consensus criteria.

In 2026, an international multi‐society consensus, endorsed by United European Gastroenterology, the European Association for the Study of Diabetes, and other leading gastroenterological and metabolic societies, established MRI‐derived proton density fat fraction (PDFF) thresholds for diagnosing and categorising fatty pancreas, explicitly acknowledging the need for validation in large prospective cohorts [14]. Whether these consensus‐based thresholds identify cardiometabolic risk at the population level remains unclear. To address this gap, we investigated the association between fatty pancreas, defined using consensus‐based MRI‐PDFF thresholds, and prevalent and incident type 2 diabetes (T2D), CKD, and major adverse cardiovascular events (MACE) in the UKBB.

## 2 Methods

### 2.1 Study Population

Data were obtained from the UKBB, a prospective population‐based cohort comprising over 500,000 participants aged 40–69 years, recruited across the United Kingdom between 2006 and 2010, with ongoing linkage to national health, death and procedural registries [15]. A subset of participants underwent abdominal MRI scanning as part of the UKBB imaging enhancement sub‐study [16]. Analyses were restricted to participants of European ancestry, who comprised approximately 70% of the imaging sub‐study, to limit confounding by population stratification and reduce heterogeneity related to ancestry‐associated differences in body‐fat distribution and cardiometabolic risk.

From participants with quantifiable pancreatic PDFF, we excluded those with a history of cancer within the 5 years preceding MRI (excluding non‐melanoma skin cancers), conditions that could affect pancreatic fat or body composition (acute or chronic pancreatitis, pancreatic cysts or pseudocysts, exocrine pancreatic insufficiency, pancreatic tumours, cystic fibrosis, lipodystrophy, malnutrition, or cachexia), or a history of pancreatic, bariatric or upper‐gastrointestinal surgery before MRI. The corresponding ICD‐10 and OPCS‐4 codes are reported in Supporting Information S1: Table S1. Participants missing BMI or MRI‐derived visceral adipose tissue (VAT), the two adiposity covariates required for the primary multivariable models, were also excluded, yielding a final analytic cohort of 19,255 participants.

### 2.2 Quantification and Categorisation of Pancreatic Fat

Pancreatic fat was quantified using MRI‐derived PDFF (UKBB Data Field 21090), obtained from a dedicated two‐dimensional, single‐slice multi‐echo gradient‐recalled echo sequence acquired at 1.5 T (Siemens Aera, Siemens Healthineers, Erlangen, Germany). Pancreatic PDFF was derived from a representative axial pancreatic slice using magnitude and phase information to jointly estimate PDFF and R2* [16]. This UKBB image‐derived phenotype reflects a slice‐based measurement of pancreatic fat rather than a whole‐organ volumetric assessment, and is distinct from the three‐dimensional, two‐point Dixon sequence used for volumetric organ segmentation. Participants were categorised according to the 2026 international consensus thresholds as follows: < 6% (normal), 6 to < 16% (mild fatty pancreas), and ≥ 16% (moderate‐to‐severe fatty pancreas) [14]. Pancreatic PDFF was also analysed as a continuous variable, with effect estimates expressed per 5% increment.

### 2.3 Outcomes

The primary outcomes were prevalent and incident T2D, CKD, and MACE ascertained through linkage to hospital inpatient records, death registries, and procedural registries using International Classification of Diseases, 10th Revision (ICD‐10) and Office of Population Censuses and Surveys Classification of Interventions and Procedures, version 4 (OPCS‐4) codes. The codes used to define each outcome are reported in Supporting Information S1: Table S2. Prevalent outcomes were defined as those recorded at or before the MRI scan, whereas incident outcomes were defined as the first occurrence after MRI. Prevalent T2D was identified using ICD‐10 codes E11 or E14, self‐reported diagnosis (UKBB field 1223), or use of glucose‐lowering medication, with exclusion of type 1 diabetes (E10); incident T2D was defined as the first hospital‐recorded ICD‐10 code E11 or E14 after MRI. CKD was defined using the ICD‐10 code N18 and renal replacement‐related OPCS‐4 procedure codes for both prevalent and incident analyses. MACE was a composite of ischaemic heart disease, ischaemic stroke, transient ischaemic attack, and coronary revascularisation; incident MACE additionally included cardiovascular death, defined using the same set of ICD‐10 codes. Follow‐up time was calculated from the MRI scan, which served as the analytical baseline, to the first event, death, or censoring on March 31, 2023, whichever occurred first.

### 2.4 Statistical Analysis

Baseline characteristics were summarised as median [interquartile range] for continuous variables and as frequency (percentage) for categorical variables. Differences across pancreatic PDFF categories were assessed using the Kruskal‐Wallis test for continuous variables and the chi‐squared or Fisher's exact test for categorical variables, as appropriate. Associations with prevalent outcomes were estimated using multivariable logistic regression and reported as odds ratios (ORs) with 95% confidence intervals (CIs). Incident outcomes were analysed using Cox proportional hazard models and reported as hazard ratios (HRs) with 95% CIs. For incident analyses, participants with the prevalent outcome at or before the MRI were excluded from the corresponding risk set.

Pancreatic fat categories were modelled with the < 6% group as the reference category. All primary models were adjusted for age, sex, BMI‐defined obesity (BMI ≥ 30 kg/m^2^), and elevated MRI‐derived VAT (≥ the cohort‐specific 90th percentile), all assessed at the imaging visit. These adiposity covariates were modelled categorically to capture clinically meaningful phenotypes and maintain consistency with the other binary cardiometabolic risk factors. Models for CKD were additionally adjusted for T2D and hypertension, whereas models for MACE were further adjusted for T2D, hypertension, dyslipidaemia, and smoking status at the imaging visit. The definitions and timing of these comorbidities are detailed in Supporting Information S1: Table S3. Trend analyses were performed by modelling pancreatic fat categories as an ordinal variable in both logistic and Cox regression models, with *p* for trend reported for each outcome. The proportional hazard assumption and collinearity among pancreatic PDFF categories, BMI‐defined obesity, and elevated MRI‐derived VAT were assessed as detailed in the Supplementary Methods.

Several sensitivity analyses were performed. To assess robustness to confounding factors, the primary models were additionally adjusted for alcohol intake at the imaging visit, expressed as grams/day and derived from UKBB touchscreen questionnaire data as detailed in the Supporting Information S1: Methods, and for MRI‐derived liver PDFF, both modelled continuously in separate models. The primary models were also re‐run adjusting for obesity and elevated VAT separately rather than jointly. To assess robustness to outcome definition, the T2D definition was restricted to ICD‐10 code E11, excluding E14. To address potential reverse causation, incident analyses were repeated after excluding events occurring within the first year after MRI. To account for the competing risk of death, Fine‐Grey subdistribution hazard models were fitted for incident CKD and non‐fatal MACE. For CKD, death from any cause was treated as a competing event. Because cardiovascular death was part of the main MACE composite, the competing‐risk analysis for MACE was restricted to non‐fatal MACE, with non‐cardiovascular death treated as the competing event. Finally, exploratory analyses examined the individual incident components of the MACE composite. All analyses used a complete‐case approach and were performed using R version 4.5.2.

### 2.5 Ethics

UKBB received ethical approval from the North West Multi‐Centre Research Ethics Committee (reference 21/NW/0157), and all participants provided written informed consent. This analysis was conducted under UKBB application number 37142.

## 3 Results

### 3.1 Cohort Characteristics

**FIGURE 1:**
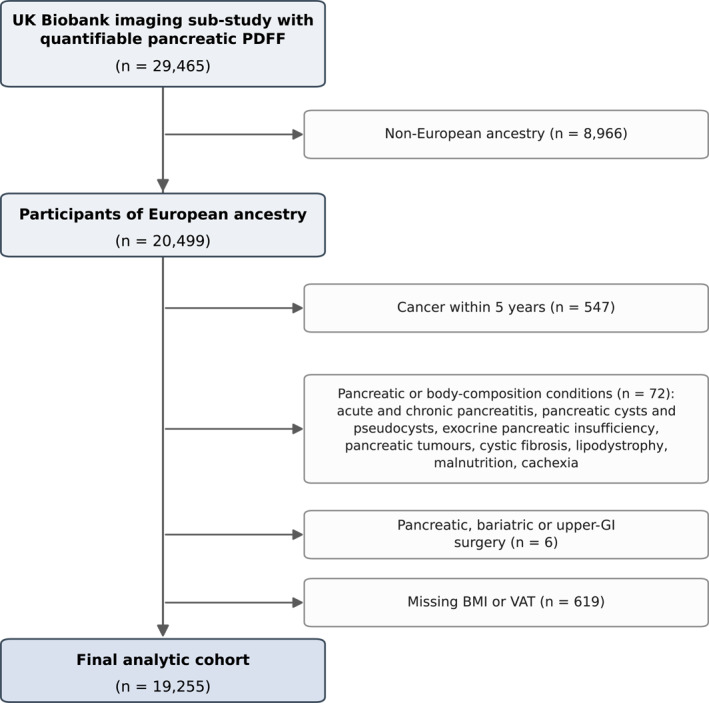
Flowchart of participant selection from the UK Biobank. The analytic cohort was drawn from participants of the UK Biobank imaging sub‐study with quantifiable pancreatic PDFF. Pancreatic or body‐composition conditions, pancreatic tumours, and pancreatic, bariatric or upper‐gastrointestinal surgery were ascertained from hospital inpatient and procedural records; the corresponding ICD‐10 and OPCS‐4 codes are reported in Supporting Information S1: Table S1. Participants missing body mass index (BMI) or visceral adipose tissue (VAT) were excluded to yield a complete‐case analytic cohort. Exclusions were applied sequentially, and participants meeting more than one criterion were counted only once at the first applicable step. BMI, body mass index; PDFF, proton density fat fraction; VAT, visceral adipose tissue; GI, gastrointestinal.

Among 19,255 UKBB participants who met the eligibility criteria (Figure 1), the median age was 65 years (IQR 58–70) and 49.1% were men. The median pancreatic PDFF was 8.1% (IQR 4.9–13.7); 6679 participants (34.7%) had pancreatic PDFF < 6% (normal), 8886 (46.1%) had PDFF 6 to < 16% (mild fatty pancreas), and 3690 (19.2%) had PDFF ≥ 16% (moderate‐to‐severe fatty pancreas) (Table 1). Moderate‐to‐severe fatty pancreas was more common in men than in women (68.6% vs. 31.4%). Higher pancreatic PDFF categories were characterised by stepwise increase in VAT (1.82, 3.86, and 5.51 L), BMI (23.6, 26.6, and 28.5 kg/m^2^), waist circumference (79, 90.6, and 97 cm), and liver PDFF (2.2%, 3.5%, and 4.9%), as well as by higher prevalence of T2D (1.2%, 4.4%, and 8.6%), hypertension (17.5%, 30.8%, and 41.7%), and dyslipidaemia (14.4%, 27.2%, and 38.2%) across pancreatic PDFF categories (all *p* < 0.001).

**TABLE 1:** Baseline characteristics of the study population according to pancreatic fat categories defined by MRI‐derived proton density fat fraction (PDFF).

	Overall	Normal pancreas (< 6%)	Mild fatty pancreas (6 to < 16%)	Moderate‐to‐severe fatty pancreas (≥ 16%)	*p*‐value
*N*	19,255	6679 (34.7%)	8886 (46.1%)	3690 (19.2%)	
Age, years	65.00 [58.00–70.00]	62.00 [56.00–68.00]	65.00 [59.00–70.00]	68.00 [62.00–72.00]	< 0.001
Men, *n* (%)	9463 (49.1%)	1955 (29.3%)	4976 (56.0%)	2532 (68.6%)	< 0.001
BMI, kg/m^2^	25.84 [23.48–28.76]	23.61 [21.84–25.77]	26.56 [24.41–29.22]	28.52 [26.03–31.37]	< 0.001
Waist circumference, cm	88.00 [79.00–97.00]	79.00 [73.00–86.00]	90.60 [83.00–98.00]	97.00 [90.00–104.00]	< 0.001
Obesity (BMI ≥ 30 kg/m^2^), *n* (%)	3421 (17.8%)	314 (4.7%)	1782 (20.1%)	1325 (35.9%)	< 0.001
Pancreatic PDFF, %	8.08 [4.85–13.72]	4.06 [3.11–4.96]	9.38 [7.51–12.02]	22.59 [18.72–28.25]	< 0.001
Liver PDFF, %	3.10 [2.20–5.40]	2.20 [1.80–3.10]	3.50 [2.50–6.20]	4.90 [3.20–9.20]	< 0.001
MRI‐derived VAT, L	3.32 [1.98–5.12]	1.82 [1.21–2.67]	3.86 [2.67–5.32]	5.51 [4.07–7.09]	< 0.001
Ever smoker, *n* (%)	6890 (35.8%)	1971 (29.5%)	3383 (38.1%)	1536 (41.6%)	< 0.001
Alcohol intake, g/day	10.29 [2.89–18.86]	8.57 [2.37–16.00]	10.29 [3.42–20.57]	10.29 [2.76–20.57]	< 0.001
Hypertension, *n* (%)	5440 (28.3%)	1170 (17.5%)	2733 (30.8%)	1537 (41.7%)	< 0.001
Dyslipidaemia, *n* (%)	4791 (24.9%)	965 (14.4%)	2418 (27.2%)	1408 (38.2%)	< 0.001
Type 2 diabetes, *n* (%)	791 (4.1%)	83 (1.2%)	390 (4.4%)	318 (8.6%)	< 0.001

*Note:* Pancreatic fat categories were defined according to consensus‐based PDFF thresholds as < 6% (normal), 6 to < 16% (mild fatty pancreas), and ≥ 16% (moderate‐to‐severe fatty pancreas). Continuous variables are presented as median [interquartile range (IQR)], and categorical variables as *n* (%). Differences across groups were assessed using the Kruskal‐Wallis test for continuous variables and the chi‐square or Fisher's exact test for categorical variables, as appropriate. Anthropometric measures, MRI‐derived adiposity measures, alcohol intake, smoking status, and medication use were assessed at the imaging visit. Prevalent comorbidities were defined using hospital inpatient diagnoses recorded up to the imaging visit, self‐reported diagnoses from the recruitment assessment, and medication use at the imaging visit. Detailed definitions of comorbidities are provided in Supporting Information S1: Table S3.

Abbreviations: BMI, body mass index; PDFF, proton density fat fraction; VAT, visceral adipose tissue.

### 3.2 Fatty Pancreas and Cardiometabolic Outcomes

Cross‐sectional analyses showed a graded association between fatty pancreas and prevalent T2D. Compared with participants with pancreatic PDFF < 6%, the adjusted ORs (aORs) for prevalent T2D were 2.37 (95% CI 1.86–3.06; *p* < 0.001) for mild fatty pancreas and 3.25 (95% CI 2.49–4.27; *p* < 0.001) for moderate‐to‐severe fatty pancreas (*p* for trend < 0.001). In longitudinal analyses, after exclusion of participants with prevalent T2D at or before MRI, the adjusted HRs (aHRs) for incident T2D were 2.19 (95% CI 1.40–3.42; *p* < 0.001) and 2.72 (95% CI 1.66–4.46; *p* < 0.001) for mild and moderate‐to‐severe fatty pancreas, respectively (*p* for trend < 0.001). When pancreatic PDFF was modelled continuously, each 5% increase was associated with higher odds and hazard of T2D (OR 1.16, 95% CI 1.12–1.21; HR 1.17, 95% CI 1.09–1.25; both *p* < 0.001).

For CKD, no independent association was observed in cross‐sectional analyses (aOR 1.75, 95% CI 0.99–3.21; *p* = 0.059 for mild fatty pancreas, and aOR 1.42, 95% CI 0.73–2.83; *p* = 0.304 for moderate‐to‐severe fatty pancreas; *p* for trend = 0.456). In contrast, fatty pancreas was associated with incident CKD, particularly in the moderate‐to‐severe category. The aHRs were 1.28 (95% CI 0.93–1.75; *p* = 0.124) for mild fatty pancreas and 1.82 (95% CI 1.29–2.57; *p* < 0.001) for moderate‐to‐severe fatty pancreas (*p* for trend < 0.001). Each 5% increase in pancreatic PDFF was associated with an 11% higher hazard of incident CKD (HR 1.11, 95% CI 1.05–1.18; *p* < 0.001).

For MACE, cross‐sectional analyses showed an association for moderate‐to‐severe fatty pancreas only (aOR 1.26, 95% CI 1.04–1.56; *p* = 0.022), with no association for mild fatty pancreas (aOR 1.09, 95% CI 0.92–1.32; *p* = 0.289; *p* for trend = 0.017). In longitudinal analyses, both fatty pancreas categories were associated with a modest increase in incident MACE risk, with aHRs of 1.29 (95% CI 1.06–1.59; *p* = 0.013) for mild fatty pancreas and 1.30 (95% CI 1.02–1.66; *p* = 0.034) for moderate‐to‐severe fatty pancreas (*p* for trend = 0.041). When pancreatic PDFF was modelled continuously, each 5% increase was associated with higher odds of prevalent MACE (OR 1.05, 95% CI 1.01–1.09; *p* = 0.006), but not with incident MACE (HR 1.02, 95% CI 0.98–1.07; *p* = 0.323). Exploratory analyses of individual incident MACE components are reported in Supporting Information S1: Table S4.

**FIGURE 2:**
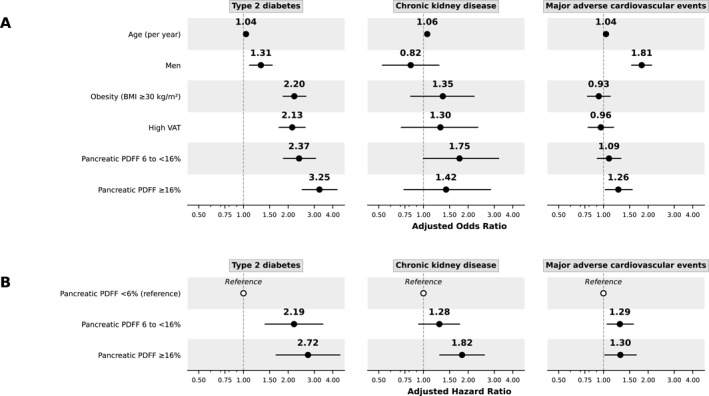
Association between fatty pancreas and type 2 diabetes, chronic kidney disease (CKD), and major adverse cardiovascular events (MACE). (A) Adjusted odds ratios (ORs) for prevalent outcomes. (B) Adjusted hazard ratios (HRs) for incident outcomes. Fatty pancreas was categorised using MRI‐derived proton density fat fraction (PDFF) thresholds as < 6% (reference), 6 to < 16% (mild fatty pancreas), and ≥ 16% (moderate‐to‐severe fatty pancreas). For clarity, only estimates for fatty pancreas categories and covariates common to all outcomes are shown (age, sex, BMI‐defined obesity [BMI ≥ 30 kg/m^2^], and elevated MRI‐derived visceral adipose tissue [VAT, ≥ 90th percentile]). Additional adjustments were applied as follows: models for CKD were further adjusted for type 2 diabetes and hypertension, and models for MACE were further adjusted for type 2 diabetes, hypertension, dyslipidaemia, and smoking status. Error bars indicate 95% confidence intervals.

Median follow‐up was similar across pancreatic PDFF categories within each outcome‐specific risk set, ranging from 4.77 to 4.82 years across categories and outcomes; corresponding values were 4.80–4.82 years for T2D, 4.79–4.81 years for CKD, and 4.77 years for MACE. Complete estimates are shown in Table 2 and Figure 2.

**TABLE 2:** Association between fatty pancreas and outcomes.

	Pancreatic PDFF	Cases (prevalent), *n* (%)	Adjusted OR (95% CI)	*p*‐value	Events (incident), *n* (%)	Adjusted HR (95% CI)	*p*‐value
Type 2 diabetes	< 6%	83 (1.2%)	Ref.	—	26 (0.4%)	Ref.	—
	6 to < 16%	390 (4.4%)	2.37 (1.86–3.06)	< 0.001	108 (1.3%)	2.19 (1.40–3.42)	< 0.001
	≥ 16%	318 (8.6%)	3.25 (2.49–4.27)	< 0.001	77 (2.3%)	2.72 (1.66–4.46)	< 0.001
	P for trend	—	—	< 0.001	—	—	< 0.001
	Per 5% increase in PDFF	—	1.16 (1.12–1.21)	< 0.001	—	1.17 (1.09–1.25)	< 0.001
Chronic kidney disease	< 6%	16 (0.2%)	Ref.	—	58 (0.9%)	Ref.	—
	6 to < 16%	59 (0.7%)	1.75 (0.99–3.21)	0.059	150 (1.7%)	1.28 (0.93–1.75)	0.124
	≥ 16%	30 (0.8%)	1.42 (0.73–2.83)	0.304	130 (3.6%)	1.82 (1.29–2.57)	< 0.001
	P for trend	—	—	0.456	—	—	< 0.001
	Per 5% increase in PDFF	—	1.05 (0.93–1.16)	0.424	—	1.11 (1.05–1.18)	< 0.001
MACE	< 6%	203 (3.0%)	Ref.	—	144 (2.2%)	Ref.	—
	6 to < 16%	577 (6.5%)	1.09 (0.92–1.32)	0.289	346 (4.2%)	1.29 (1.06–1.59)	0.013
	≥ 16%	392 (10.6%)	1.26 (1.04–1.56)	0.022	185 (5.7%)	1.30 (1.02–1.66)	0.034
	P for trend	—	—	0.017	—	—	0.041
	Per 5% increase in PDFF	—	1.05 (1.01–1.09)	0.006	—	1.02 (0.98–1.07)	0.323

*Note:* Fatty pancreas was categorised according to MRI‐derived pancreatic PDFF thresholds as < 6%, 6 to < 16%, and ≥ 16%. Prevalent outcomes were assessed at or before MRI, and associations were estimated using multivariable logistic regression models, reported as odds ratios (ORs) with 95% confidence intervals (CIs). Incident outcomes were defined as the first occurrence after MRI, with exclusion of participants with the corresponding prevalent disease at the time of MRI, and were analysed using Cox proportional hazards models, reported as hazard ratios (HRs) with 95% CIs. Median follow‐up was 4.8 years (IQR 4.1–5.7). Participants were followed from MRI to first outcome, death, or censoring on March 31, 2023. All analyses were conducted in the complete‐case analytic cohort. All models were adjusted for age, sex, BMI‐defined obesity (BMI ≥ 30 kg/m^2^), and elevated MRI‐derived VAT (≥ the cohort‐specific 90th percentile), all assessed at the imaging visit. Models for CKD were additionally adjusted for type 2 diabetes and hypertension; models for MACE were additionally adjusted for type 2 diabetes, hypertension, dyslipidaemia, and smoking status. Continuous analyses were performed per 5% increment in pancreatic PDFF. P for trend was calculated by modelling fatty pancreas categories as an ordinal variable.

Abbreviations: BMI, body mass index; CI, confidence interval; CKD, chronic kidney disease; HR, hazard ratio; IQR, interquartile range; MACE, major adverse cardiovascular events; MRI, magnetic resonance imaging; OR, odds ratio; PDFF, proton density fat fraction; VAT, visceral adipose tissue.

The principal findings for T2D and CKD were consistent across sensitivity analyses. Associations were preserved after additional adjustment for alcohol intake and, separately, for MRI‐derived liver PDFF, although T2D estimates were attenuated after liver PDFF adjustment. Results were also consistent when obesity and elevated VAT were used as alternative adiposity adjustments, when the T2D definition was restricted to ICD‐10 code E11, and after exclusion of events occurring within the first year after MRI (Supporting Information S1: Tables S5–S9). Competing‐risk models accounting for death yielded similar findings for incident CKD and non‐fatal MACE (Supporting Information S1: Table S10). The association with incident MACE, which was modest in the primary analysis, was preserved in most sensitivity analyses but was attenuated after exclusion of first‐year events.

## 4 Discussion

In this large population‐based cohort applying the 2026 international multidisciplinary consensus‐derived MRI PDFF thresholds, fatty pancreas was independently associated with prevalent and incident T2D. This association persisted after adjustment for age, sex, BMI‐defined obesity and elevated MRI‐derived VAT, with a continuous dose‐response relationship when pancreatic PDFF was modelled per 5% increment. These findings are consistent with prior prospective evidence linking intrapancreatic fat to the onset of T2D [8, 9]. However, previous studies defined fatty pancreas using empirically derived or study‐specific thresholds, such as sex‐ and age‐specific cut‐offs, rather than standardised, consensus‐based criteria. The observed association is biologically plausible, given the established link between fatty pancreas and beta cell dysfunction, whereby lipotoxic and inflammatory effects on pancreatic islets may impair insulin secretion, promote insulin resistance, and contribute to beta cell dedifferentiation [4, 5, 6].

Fatty pancreas was also independently associated with incident CKD, particularly in the moderate‐to‐severe category, with the continuous analysis further supporting this link. This association was not observed in cross‐sectional analyses. While this pattern may suggest that renal injury develops progressively over time, potentially consistent with lipotoxic mechanisms of renal damage, alternative explanations cannot be excluded, including limited statistical power for prevalent CKD, underdiagnosis of renal impairment in administrative records, and residual confounding [11, 12]. A time‐dependent effect is biologically plausible, as free fatty acids and their metabolites induce endoplasmic reticulum stress, mitochondrial dysfunction, oxidative stress, and inflammation, which may progressively damage glomerular and tubular cells [17]. However, this interpretation requires confirmation in studies with longer follow‐up and direct measures of renal function.

Although associations with MACE were present, they were modest and lacked a clear dose‐response gradient across both prevalent and incident analyses. Consistent with this, the association with incident MACE was attenuated after excluding events occurring within the first year after MRI, indicating that it should be interpreted with caution. These findings are broadly consistent with prior evidence linking intrapancreatic fat to cardiovascular risk and suggest that fatty pancreas may reflect a broader cardiometabolic risk phenotype, mediated through shared pathways including dyslipidaemia, endothelial dysfunction, and systemic inflammation, rather than acting as a primary and independent cardiovascular risk driver [12].

Together, these findings support fatty pancreas as a clinically relevant component of the cardiovascular‐kidney‐metabolic continuum [18, 19]. To our knowledge, this is one of the first large‐scale prospective studies to apply the MRI‐derived PDFF thresholds proposed by the 2026 international consensus for fatty pancreas. Our results provide population‐level outcome‐based support for these criteria, particularly for T2D and incident CKD [14]. The associations were robust across multiple sensitivity analyses, including adjustment for alcohol intake and liver PDFF, restriction of T2D to ICD‐10 code E11, exclusion of early events, and competing‐risk modelling. Overall, these results suggest that pancreatic PDFF may capture aspects of ectopic fat‐related metabolic vulnerability not fully reflected by traditional clinical variables. However, pancreatic PDFF should not be interpreted as a standalone risk stratification tool. Rather, pancreatic fat quantification may provide complementary information within an integrated cardiometabolic risk assessment approach, particularly in people undergoing abdominal MRI for clinical or research indications. Further studies should determine whether changes in pancreatic PDFF track changes in cardiometabolic risk and whether interventions such as lifestyle modification, weight loss, incretin‐based therapies, or other metabolic treatments can reduce pancreatic fat and modify the associated risk. Future work integrating pancreatic fat with pancreatic volume may also refine risk assessment [20].

Several limitations warrant consideration. First, the observational design precludes causal inference. Second, pancreatic PDFF was derived from a slice‐based MRI measurement rather than a whole‐organ volumetric assessment, which may not fully capture regional heterogeneity of pancreatic fat, and its assessment at a single‐time‐point precluded the evaluation of longitudinal changes in fatty pancreas. Third, outcomes and exclusion criteria were ascertained from administrative ICD‐10 and OPCS‐4 codes, which may have led to misclassification or underascertainment, including incomplete capture of conditions potentially affecting pancreatic morphology. Glycated haemoglobin and estimated glomerular filtration rate were not available at the imaging visit, which may have led to underascertainment of T2D and CKD. Therefore, residual confounding by incompletely ascertained pancreatic disorders cannot be excluded. Finally, the characteristics of the study population may affect the interpretation and generalisability of our findings. The UKBB imaging sub‐study is subject to healthy volunteer bias, which may have led to underestimation of true associations in the general population. In addition, participants were predominantly middle‐aged or older adults; therefore, findings may not be generalisable to younger populations, and the high prevalence of fatty pancreas observed in our cohort may partly reflect the age‐related increase in intrapancreatic fat [21]. Analyses were also restricted to participants of European ancestry to limit confounding by population stratification; therefore, validation in ancestrally diverse cohorts is warranted.

In conclusion, fatty pancreas, defined using the 2026 consensus MRI‐derived PDFF thresholds, was independently associated with prevalent and incident T2D, and incident CKD, with more modest associations observed for MACE. The strongest and most consistent associations were observed for moderate‐to‐severe fatty pancreas, particularly for T2D and CKD. These findings support the clinical relevance of consensus‐based PDFF thresholds within integrated cardiometabolic risk assessment in European‐ancestry populations.

## Author Contributions

**Nicola Pugliese:** conceptualization, data curation, formal analysis, writing – original draft. **Oveis Jamialahmadi:** conceptualization, data curation, supervision. **Arturo Cesaro:** conceptualization, writing – review and editing. **Valentina Flagiello:** conceptualization, writing – review and editing. **Rosellina M. Mancina:** writing – review and editing. **Alessio Aghemo:** writing – review and editing. **Stefano Romeo:** conceptualization, supervision, writing – review and editing. All authors: data interpretation, writing – review and editing, and approval of the final manuscript.

## Funding

S.R. was supported by the Region Stockholm (ALF project grant, FoUI‐1021801), the Swedish Cancerfonden (22 2270 Pj), the Swedish Research Council (Vetenskapsradet (VR), 2023‐02079), the Swedish Heart Lung Foundation (20220334), the Novo Nordisk Distinguished Investigator Grant—Endocrinology and Metabolism (NNF23OC0082114), the Novo Nordisk Project grants in Endocrinology and Metabolism (NNF24OC0091535), and a Novo Nordisk donation to the Karolinska Institutet in connection with the professor appointment.

## Conflicts of Interest

N.P. served as a speaker or advisor for Gilead, Gore, Astra Zeneca, AlfaSigma and Novo Nordisk. He also received travel grants from Gilead, AlfaSigma and AbbVie. AC has served as a member of Advisory Boards for Amgen, Sanofi, Novartis, Daiichi Sankyo, AstraZeneca, Boehringer Ingelheim, Novo Nordisk, Eli Lilly, Chiesi, Sharper, Ultragenyx, and Amarin. He has also received lecture fees from Novo Nordisk and support for attending meetings from Menarini, Daiichi Sankyo, and Novartis. AA has received speaking fees from MSD, Gilead, AlfaSigma, Intercept, AbbVie, and Sobi; has served as a consultant for Gilead, Intercept, Sobi, AbbVie, and MSD; and has received research grants from Gilead and AbbVie. In the last 5 years SR received research grants from Novo Nordisk and AstraZeneca for basic science research on steatotic liver disease, and has been consulting for AstraZeneca, GSK, Celgene Corporation, Ribocure AB, Madrigal, Ultragenyx, Amgen, Sanofi, Wave Life Sciences, Lipigon, Novartis, Profluent, Aina, Echosense and Chiesi; declares equity from Heptabio; and is inventor on a Patent with title ‘Method for treating fatty liver disease’, on PSD3, US application number 17,480266 filed on 21st September 2021. No COIs were declared by other authors.

## Supporting information


Supporting Information S1

## Data Availability

The data used in this study are available from the UK Biobank (www.ukbiobank.ac.uk) upon application and approval. Due to data access restrictions imposed by UK Biobank, the dataset cannot be made publicly available. Analytical methods and code used in this study are available from the corresponding author upon reasonable request.
