# Supplementary material for: Fatty Pancreas and Risk of Type 2 Diabetes, Chronic Kidney Disease and Cardiovascular Events: Evidence From a Population‐Based Cohort
Source: United European Gastroenterol J. 2026 Jul 2;14(6):e70253. doi: 10.1002/ueg2.70253 (PMC13326817; doi:10.1002/ueg2.70253)
Supplement: Supplementary file 1 — Supporting Information S1 [file UEG2-14-e70253-s001.docx]

**Supplementary Methods**

**Assessment of model assumptions and collinearity diagnostics**

The proportional hazards assumption was assessed for the main Cox proportional hazards models using Schoenfeld residuals. No evidence of violation was observed for pancreatic PDFF categories or for the global model tests for incident type 2 diabetes (T2D), chronic kidney disease (CKD), or major adverse cardiovascular events (MACE). The global p-values were 0.658 for incident T2D, 0.799 for incident CKD, and 0.690 for incident MACE.

Given the expected relationship between pancreatic fat, BMI-defined obesity, and visceral adipose tissue (VAT), we assessed potential collinearity among the exposure and adiposity covariates included in the primary multivariable models. Spearman correlation coefficients were calculated between continuous pancreatic PDFF, BMI, and MRI-derived VAT. Variance inflation factor diagnostics were then performed for the covariates included in the primary multivariable models. For categorical variables with more than two levels, GVIF values adjusted for degrees of freedom were calculated.

Pancreatic PDFF was moderately correlated with BMI and MRI-derived VAT when analysed continuously (Spearman rho = 0.51 for pancreatic PDFF and BMI; rho = 0.68 for pancreatic PDFF and VAT; rho = 0.74 for BMI and VAT; all p<0.001). However, no evidence of problematic multicollinearity was observed in the primary multivariable models. The maximum adjusted GVIF values were 1.17 in the base model, 1.18 in the CKD model, and 1.18 in the MACE model.

**Alcohol intake assessment and derivation**

Alcohol intake was derived from UKBB touchscreen questionnaire data collected at the imaging visit (instance 2). We used alcohol intake frequency (data-field 1558) together with beverage-specific alcohol intake fields, including average weekly intake of red wine (data-field 1568), champagne/white wine (data-field 1578), beer/cider (data-field 1588), spirits (data-field 1598), fortified wine (data-field 1608), and other alcoholic drinks (data-field 5364). When weekly intake data were unavailable or coded as “do not know” or “prefer not to answer”, average monthly intake fields were used when available, including red wine (data-field 4407), champagne/white wine (data-field 4418), beer/cider (data-field 4429), spirits (data-field 4440), fortified wine (data-field 4451), and other alcoholic drinks (data-field 4462).

Beverage-specific intake was converted into UK alcohol units using standard UK assumptions, based on UK National Health Service alcohol-unit guidance and adapted from a previously published UK Biobank alcohol-intake derivation pipeline [1]. Specifically, we assumed 1.5 units per glass of red wine, 1.5 units per glass of champagne/white wine, 2 units per pint of beer/cider, 1 unit per measure of spirits, 1.5 units per glass of fortified wine, and 1.5 units per glass of other alcoholic drinks. Total alcohol units were summed across beverage types and converted to grams of ethanol using the conversion of 1 UK alcohol unit = 8 g ethanol. Weekly intake was divided by 7 and monthly intake by 30.4375 to obtain average alcohol intake in grams/day.

Participants who reported consuming alcohol “never” or on “special occasions only” were assigned an alcohol intake of 0 g/day. Participants who reported regular alcohol intake but had 0 g/day across all beverage-specific intake fields were excluded from the derivation of alcohol intake. Participants with missing alcohol intake data after these steps were excluded from the alcohol-adjusted sensitivity analyses.

# [1] Tavaglione F, De Vincentis A, Jamialahmadi O, et al. Inborn and acquired risk factors for severe liver disease in Europeans with type 2 diabetes from the UK Biobank. *JHEP Rep*. 2021;3(3):100262. Published 2021 Mar 2. doi:10.1016/j.jhepr.2021.100262

| **Category** | **Conditions included** | **ICD-10 / OPCS-4 codes** | **Timing** |
| --- | --- | --- | --- |
| Cancer | Any malignant neoplasm except non-melanoma skin cancer | ICD-10: C00-C97 (except C44) | Within 5 years before MRI |
| Pancreatic or body-composition conditions | Acute and chronic pancreatitis, pancreatic cysts and pseudocysts, exocrine pancreatic insufficiency, pancreatic tumours, cystic fibrosis, lipodystrophy, malnutrition, cachexia | ICD-10: K85; K86; C25; D13.6; D13.7; E84; E88.1; E43-E46; R64 | Before MRI |
| Pancreatic, bariatric or upper-gastrointestinal surgery | Pancreas transplantation, total or partial pancreatectomy, pancreaticoduodenectomy, pancreatic lesion excision/destruction, pancreatic duct procedures, pancreatic drainage, gastric resection, bypass, and related upper-gastrointestinal procedures | OPCS-4: J54-J61; G27; G28; G30; G31-G33; G38 | Before MRI |

**Supplementary Table 1. ICD-10 and OPCS-4 codes used to define exclusions for the study population in the UK Biobank.** Exclusion criteria were applied using ICD-10 diagnostic codes from hospital inpatient records, supplemented by OPCS-4 procedural codes where applicable. Codes are reported without the decimal point, as implemented in the analysis (e.g., K85 includes K85.0–K85.9). All exclusions were ascertained before the imaging visit, except for cancer, which was ascertained within the 5 years preceding the imaging visit. Abbreviations: ICD-10, International Classification of Diseases, Tenth Revision; OPCS-4, Office of Population Censuses and Surveys Classification of Interventions and Procedures, version 4; MRI, magnetic resonance imaging.

| **Outcome** | **ICD-10 / OPCS-4 codes** |
| --- | --- |
| Type 2 diabetes | ICD-10: E11; E14 (type 1 diabetes, E10, excluded) |
| Chronic kidney disease | ICD-10: N18  OPCS-4: L74.1-L74.6; L74.8; L74.9; M01.2-M01.9; M02.3; M08.4; M17.2; M17.8; M17.9; X40.2; X40.5; X40.6; X41.1; X41.2 |
| Major adverse cardiovascular events (MACE) | ICD-10: I20-I25; I63; G45  OPCS-4 (revascularisation): K40–K46; K49; K50  Cardiovascular death (ICD-10): I20-I25; I63; G45 |

**Supplementary Table 2. ICD-10 and OPCS-4 codes used to define study outcomes in the UK Biobank.** Outcomes were defined using ICD-10 diagnostic codes from hospital inpatient records and death registry data, supplemented by OPCS-4 procedural codes for coronary revascularisation. The MACE composite comprised ischaemic heart disease, ischaemic stroke, transient ischaemic attack, and coronary revascularisation; incident MACE additionally included cardiovascular death. Abbreviations: ICD-10, International Classification of Diseases, Tenth Revision; OPCS-4, Office of Population Censuses and Surveys Classification of Interventions and Procedures, version 4.

| **Comorbidity** | **Definition** |
| --- | --- |
| Hypertension | ICD-10 code I10-I13 recorded before the imaging visit; self-reported hypertension at the baseline assessment (UKBB field 20002, codes 1065 and 1072); or use of antihypertensive medication at the imaging visit (UKBB field 20003). |
| Dyslipidaemia | ICD-10 code E78 recorded before the imaging visit; self-reported dyslipidaemia at the baseline assessment (UKBB field 20002, code 1473); or use of lipid-lowering medication at the imaging visit (UKBB field 20003). |
| Type 2 diabetes | ICD-10 codes E11 or E14 recorded before the imaging visit; self-reported diabetes at the baseline assessment (UKBB field 1223); or use of glucose-lowering medication at the imaging visit (UKBB field 20003). Participants with type 1 diabetes (ICD-10 E10) were excluded. |

**Supplementary Table 3. Definitions of comorbidities used in the analysis.** Comorbidities were defined using combinations of ICD-10 diagnostic codes from hospital inpatient records recorded before the imaging visit, self-reported diagnoses at the baseline (recruitment) assessment, and medication use recorded at the imaging visit. Participants with prevalent type 1 diabetes (ICD-10 E10) before the imaging visit were excluded from the type 2 diabetes definition. Abbreviations: ICD-10, International Classification of Diseases, Tenth Revision; UKBB, UK Biobank.

| **MACE component** | **Pancreatic PDFF** | **Incident events, n (%)** | **Adjusted HR (95% CI)** | **p-value** |
| --- | --- | --- | --- | --- |
| Ischaemic heart disease / coronary revascularisation | <6% | 103 (1.6%) | Ref. | — |
|  | 6 to <16% | 273 (3.3%) | 1.39 (1.09–1.76) | 0.007 |
|  | ≥16% | 130 (3.9%) | 1.22 (0.92–1.63) | 0.174 |
|  | P for trend | — | — | 0.225 |
|  | Per 5% increase in PDFF | — | 1.00 (0.94–1.05) | 0.920 |
| Cerebrovascular events | <6% | 46 (0.7%) | Ref. | — |
|  | 6 to <16% | 84 (1.0%) | 1.02 (0.70–1.49) | 0.921 |
|  | ≥16% | 60 (1.7%) | 1.37 (0.88–2.11) | 0.160 |
|  | P for trend | — | — | 0.145 |
|  | Per 5% increase in PDFF | — | 1.07 (0.99–1.17) | 0.086 |
| Cardiovascular death | <6% | 11 (0.2%) | Ref. | — |
|  | 6 to <16% | 22 (0.2%) | 0.71 (0.34–1.52) | 0.379 |
|  | ≥16% | 27 (0.7%) | 1.15 (0.52–2.54) | 0.726 |
|  | P for trend | — | — | 0.442 |
|  | Per 5% increase in PDFF | — | 1.04 (0.90–1.19) | 0.605 |

**Supplementary Table 4. Association between pancreatic fat and individual components of the MACE composite.** Exploratory analyses evaluated the association between fatty pancreas and the individual incident components of the MACE composite. Fatty pancreas was categorised according to MRI-derived pancreatic PDFF thresholds as <6%, 6 to <16%, and ≥16%. Incident events were defined as the first occurrence after MRI, with exclusion of participants with the corresponding component before MRI, and were analysed using Cox proportional hazards models, reported as hazard ratios (HRs) with 95% confidence intervals (CIs). Participants were followed from MRI to first event, death, or censoring on March 31, 2023. All analyses were conducted in the complete-case analytic cohort. Models were adjusted for age at imaging, sex, obesity defined as BMI ≥30 kg/m² at imaging, MRI-derived VAT defined as values ≥ the cohort-specific 90th percentile, type 2 diabetes, hypertension, dyslipidaemia, and smoking status. Continuous analyses were performed per 5% increment in pancreatic PDFF. P for trend was calculated by modelling fatty pancreas categories as an ordinal variable. Abbreviations: MACE, major adverse cardiovascular events; PDFF, proton density fat fraction.

| **Outcome** | **Pancreatic PDFF** | **Prevalent,**  **OR (95% CI)** | **p** | **Incident,**  **HR (95% CI)** | **p** |
| --- | --- | --- | --- | --- | --- |
| Type 2 diabetes | <6% | Ref. | — | Ref. | — |
|  | 6 to <16% | 2.41 (1.89–3.12) | <0.001 | 2.21 (1.41–3.46) | <0.001 |
|  | ≥16% | 3.33 (2.55–4.39) | <0.001 | 2.76 (1.69–4.53) | <0.001 |
|  | P for trend | — | <0.001 | — | <0.001 |
|  | Per 5% increase in PDFF | 1.16 (1.12–1.21) | <0.001 | 1.17 (1.09–1.25) | <0.001 |
| Chronic kidney disease | <6% | Ref. | — | Ref. | — |
|  | 6 to <16% | 1.78 (1.02–3.26) | 0.051 | 1.31 (0.95–1.79) | 0.094 |
|  | ≥16% | 1.44 (0.74–2.87) | 0.288 | 1.83 (1.29–2.58) | <0.001 |
|  | P for trend | — | 0.439 | — | <0.001 |
|  | Per 5% increase in PDFF | 1.05 (0.93–1.16) | 0.427 | 1.11 (1.05–1.17) | <0.001 |
| MACE | <6% | Ref. | — | Ref. | — |
|  | 6 to <16% | 1.11 (0.92–1.33) | 0.280 | 1.29 (1.05–1.58) | 0.016 |
|  | ≥16% | 1.28 (1.04–1.57) | 0.021 | 1.30 (1.02–1.65) | 0.037 |
|  | P for trend | — | 0.017 | — | 0.045 |
|  | Per 5% increase in PDFF | 1.05 (1.01–1.09) | 0.007 | 1.02 (0.98–1.07) | 0.351 |

**Supplementary Table 5. Sensitivity analyses for prevalent and incident outcomes additionally adjusted for alcohol intake.** Fatty pancreas was categorised according to MRI-derived pancreatic PDFF thresholds as <6%, 6 to <16%, and ≥16%. Prevalent outcomes were defined at or before MRI and analysed using multivariable logistic regression (odds ratios, ORs); incident outcomes were defined as the first occurrence after MRI, with exclusion of participants with the corresponding prevalent disease at the time of MRI, and analysed using Cox proportional hazards models (hazard ratios, HRs), each with 95% confidence intervals (CIs). Participants were followed from MRI to first outcome, death, or censoring on March 31, 2023. Participants with missing alcohol intake data (n = 204) were excluded from these analyses. Models were adjusted as in the primary analyses (age at imaging, sex, obesity [BMI ≥30 kg/m²], and high MRI-derived visceral adipose tissue [≥90th percentile]; models for CKD additionally for type 2 diabetes and hypertension; models for MACE additionally for type 2 diabetes, hypertension, dyslipidaemia, and smoking status), with the addition of alcohol intake at the imaging visit, modelled continuously as grams/day. Continuous analyses were performed per 5% increment in pancreatic PDFF. P for trend was calculated by modelling pancreatic fat categories as an ordinal variable. Abbreviations: BMI, body mass index; CI, confidence interval; CKD, chronic kidney disease; HR, hazard ratio; MACE, major adverse cardiovascular events; OR, odds ratio; PDFF, proton density fat fraction; T2D, type 2 diabetes; VAT, visceral adipose tissue.

| **Outcome** | **Pancreatic PDFF** | **Prevalent,**  **OR (95% CI)** | **p** | **Incident,**  **HR (95% CI)** | **p** |
| --- | --- | --- | --- | --- | --- |
| Type 2 diabetes | <6% | Ref. | — | Ref. | — |
|  | 6 to <16% | 2.07 (1.61–2.69) | <0.001 | 1.81 (1.14–2.87) | 0.012 |
|  | ≥16% | 2.78 (2.12–3.68) | <0.001 | 2.37 (1.44–3.92) | <0.001 |
|  | P for trend | — | <0.001 | — | <0.001 |
|  | Per 5% increase in PDFF | 1.15 (1.10–1.20) | <0.001 | 1.16 (1.08–1.24) | <0.001 |
| Chronic kidney disease | <6% | Ref. | — | Ref. | — |
|  | 6 to <16% | 1.96 (1.10–3.68) | 0.028 | 1.33 (0.97–1.84) | 0.081 |
|  | ≥16% | 1.58 (0.79–3.26) | 0.202 | 1.90 (1.34–2.72) | <0.001 |
|  | P for trend | — | 0.312 | — | <0.001 |
|  | Per 5% increase in PDFF | 1.06 (0.94–1.18) | 0.326 | 1.12 (1.06–1.19) | <0.001 |
| MACE | <6% | Ref. | — | Ref. | — |
|  | 6 to <16% | 1.13 (0.94–1.36) | 0.204 | 1.30 (1.05–1.60) | 0.014 |
|  | ≥16% | 1.30 (1.05–1.62) | 0.014 | 1.29 (1.01–1.67) | 0.044 |
|  | P for trend | — | 0.012 | — | 0.052 |
|  | Per 5% increase in PDFF | 1.06 (1.02–1.10) | 0.004 | 1.02 (0.97–1.07) | 0.524 |

**Supplementary Table 6. Sensitivity analyses for prevalent and incident outcomes additionally adjusted for MRI-derived liver PDFF.** Fatty pancreas was categorised according to MRI-derived pancreatic PDFF thresholds as <6%, 6 to <16%, and ≥16%. Prevalent outcomes were defined at or before MRI and analysed using multivariable logistic regression (odds ratios, ORs); incident outcomes were defined as the first occurrence after MRI, with exclusion of participants with the corresponding prevalent disease at the time of MRI, and analysed using Cox proportional hazards models (hazard ratios, HRs), each with 95% confidence intervals (CIs). Participants were followed from MRI to first outcome, death, or censoring on March 31, 2023. All analyses were conducted in the complete-case analytic cohort. Models were adjusted as in the primary analyses (age at imaging, sex, obesity [BMI ≥30 kg/m²], and high MRI-derived visceral adipose tissue [≥90th percentile]; models for CKD additionally for type 2 diabetes and hypertension; models for MACE additionally for type 2 diabetes, hypertension, dyslipidaemia, and smoking status), with the addition of MRI-derived liver PDFF, modelled as a continuous variable. Continuous analyses were performed per 5% increment in pancreatic PDFF. P for trend was calculated by modelling pancreatic fat categories as an ordinal variable. Abbreviations: BMI, body mass index; CI, confidence interval; CKD, chronic kidney disease; HR, hazard ratio; MACE, major adverse cardiovascular events; OR, odds ratio; PDFF, proton density fat fraction; T2D, type 2 diabetes; VAT, visceral adipose tissue.

| **Outcome** | **Pancreatic PDFF** | **Cases/events, n (%)** | **Adjusted OR/HR (95% CI)** | **p-value** |
| --- | --- | --- | --- | --- |
| Prevalent T2D | <6% | 42 (0.6%) | Ref. | — |
|  | 6 to <16% | 237 (2.7%) | 2.79 (2.01–3.98) | <0.001 |
|  | ≥16% | 203 (5.5%) | 3.88 (2.72–5.64) | <0.001 |
|  | P for trend | — | — | <0.001 |
|  | Per 5% increase in PDFF | — | 1.17 (1.12–1.23) | <0.001 |
| Incident T2D | <6% | 32 (0.5%) | Ref. | — |
|  | 6 to <16% | 158 (1.8%) | 2.50 (1.69–3.72) | <0.001 |
|  | ≥16% | 124 (3.6%) | 3.28 (2.14–5.02) | <0.001 |
|  | P for trend | — | — | <0.001 |
|  | Per 5% increase in PDFF | — | 1.17 (1.10–1.24) | <0.001 |

**Supplementary Table 7. Sensitivity analysis restricting the T2D definition to ICD-10 code E11 only.** Fatty pancreas was categorised according to MRI-derived pancreatic PDFF thresholds as <6%, 6 to <16%, and ≥16%. Prevalent T2D was defined at or before MRI, and associations were estimated using multivariable logistic regression models, reported as odds ratios (ORs) with 95% confidence intervals (CIs). Incident T2D was defined as the first occurrence of ICD-10 code E11 after MRI, with exclusion of participants with ICD-10 code E11 at or before the time of MRI, and was analysed using Cox proportional hazards models, reported as hazard ratios (HRs) with 95% CIs. Participants were followed from MRI to first E11 record, death, or censoring on March 31, 2023. All analyses were conducted in the complete-case analytic cohort. Models were adjusted for age at imaging, sex, obesity defined as BMI ≥30 kg/m² at imaging, and MRI-derived VAT, defined as values ≥ the cohort-specific 90th percentile. Continuous analyses were performed per 5% increment in pancreatic PDFF. P for trend was calculated by modelling pancreatic fat categories as an ordinal variable. Abbreviations: PDFF, proton density fat fraction; T2D, type 2 diabetes.

| **Outcome** | **Pancreatic PDFF** | **Obesity only,**  **OR/HR (95% CI)** | **p** | **High VAT only,**  **OR/HR (95% CI)** | **p** |
| --- | --- | --- | --- | --- | --- |
| Prevalent T2D | <6% | Ref. | — | Ref. | — |
|  | 6 to <16% | 2.35 (1.85–3.04) | <0.001 | 2.78 (2.18–3.58) | <0.001 |
|  | ≥16% | 3.55 (2.73–4.65) | <0.001 | 4.05 (3.11–5.30) | <0.001 |
|  | P for trend | — | <0.001 | — | <0.001 |
|  | Per 5% increase in PDFF | 1.19 (1.15–1.24) | <0.001 | 1.20 (1.16–1.25) | <0.001 |
| Incident T2D | <6% | Ref. | — | Ref. | — |
|  | 6 to <16% | 2.16 (1.39–3.38) | <0.001 | 2.62 (1.69–4.07) | <0.001 |
|  | ≥16% | 2.90 (1.78–4.72) | <0.001 | 3.49 (2.15–5.68) | <0.001 |
|  | P for trend | — | <0.001 | — | <0.001 |
|  | Per 5% increase in PDFF | 1.19 (1.11–1.27) | <0.001 | 1.21 (1.13–1.29) | <0.001 |
| Prevalent CKD | <6% | Ref. | — | Ref. | — |
|  | 6 to <16% | 1.74 (1.00–3.20) | 0.060 | 1.84 (1.06–3.35) | 0.038 |
|  | ≥16% | 1.46 (0.76–2.90) | 0.264 | 1.53 (0.80–3.02) | 0.206 |
|  | P for trend | — | 0.404 | — | 0.307 |
|  | Per 5% increase in PDFF | 1.05 (0.94–1.17) | 0.353 | 1.06 (0.95–1.18) | 0.296 |
| Incident CKD | <6% | Ref. | — | Ref. | — |
|  | 6 to <16% | 1.27 (0.93–1.75) | 0.130 | 1.32 (0.96–1.80) | 0.083 |
|  | ≥16% | 1.87 (1.33–2.63) | <0.001 | 1.90 (1.35–2.67) | <0.001 |
|  | P for trend | — | <0.001 | — | <0.001 |
|  | Per 5% increase in PDFF | 1.12 (1.06–1.18) | <0.001 | 1.12 (1.06–1.18) | <0.001 |
| Prevalent MACE | <6% | Ref. | — | Ref. | — |
|  | 6 to <16% | 1.09 (0.91–1.31) | 0.336 | 1.08 (0.91–1.30) | 0.384 |
|  | ≥16% | 1.26 (1.03–1.54) | 0.028 | 1.25 (1.02–1.53) | 0.034 |
|  | P for trend | — | 0.021 | — | 0.026 |
|  | Per 5% increase in PDFF | 1.05 (1.01–1.09) | 0.008 | 1.05 (1.01–1.09) | 0.009 |
| Incident MACE | <6% | Ref. | — | Ref. | — |
|  | 6 to <16% | 1.29 (1.05–1.59) | 0.013 | 1.31 (1.07–1.60) | 0.009 |
|  | ≥16% | 1.33 (1.05–1.69) | 0.020 | 1.32 (1.04–1.68) | 0.022 |
|  | P for trend | — | 0.025 | — | 0.026 |
|  | Per 5% increase in PDFF | 1.03 (0.98–1.08) | 0.200 | 1.03 (0.98–1.07) | 0.246 |

**Supplementary Table 8. Sensitivity analyses using separate adiposity adjustments (obesity or high visceral adipose tissue).** In separate models, the primary analyses were adjusted for obesity (BMI ≥30 kg/m²) alone (“Obesity only”) or for high visceral adipose tissue (VAT ≥90th percentile) alone (“VAT only”), rather than for both jointly. Fatty pancreas was categorised according to MRI-derived pancreatic PDFF thresholds as <6%, 6 to <16%, and ≥16%. Prevalent outcomes were analysed using multivariable logistic regression (odds ratios, ORs) and incident outcomes using Cox proportional hazards models (hazard ratios, HRs), each with 95% confidence intervals (CIs). Both models were additionally adjusted for age and sex; models for CKD were further adjusted for type 2 diabetes and hypertension, and models for MACE for type 2 diabetes, hypertension, dyslipidaemia, and smoking status. Continuous analyses were performed per 5% increment in pancreatic PDFF. P for trend was calculated by modelling pancreatic fat categories as an ordinal variable. All analyses were conducted in the complete-case analytic cohort. Abbreviations: BMI, body mass index; CI, confidence interval; CKD, chronic kidney disease; HR, hazard ratio; MACE, major adverse cardiovascular events; OR, odds ratio; PDFF, proton density fat fraction; T2D, type 2 diabetes; VAT, visceral adipose tissue.

| **Outcome** | **Pancreatic PDFF** | **Incident events, n (%)** | **Adjusted HR (95% CI)** | **p-value** |
| --- | --- | --- | --- | --- |
| Incident T2D | <6% | 20 (0.3%) | Ref. | — |
|  | 6 to <16% | 92 (1.1%) | 2.37 (1.43–3.92) | <0.001 |
|  | ≥16% | 65 (1.9%) | 2.90 (1.67–5.05) | <0.001 |
|  | P for trend | — | — | <0.001 |
|  | Per 5% increase in PDFF | — | 1.15 (1.06–1.24) | <0.001 |
| Incident CKD | <6% | 51 (0.8%) | Ref. | — |
|  | 6 to <16% | 127 (1.4%) | 1.29 (0.92–1.80) | 0.143 |
|  | ≥16% | 116 (3.2%) | 1.97 (1.37–2.85) | <0.001 |
|  | P for trend | — | — | <0.001 |
|  | Per 5% increase in PDFF | — | 1.13 (1.06–1.20) | <0.001 |
| Incident MACE | <6% | 125 (2.0%) | Ref. | — |
|  | 6 to <16% | 290 (3.5%) | 1.26 (1.01–1.57) | 0.040 |
|  | ≥16% | 149 (4.6%) | 1.21 (0.93–1.58) | 0.165 |
|  | P for trend | — | — | 0.185 |
|  | Per 5% increase in PDFF | — | 1.01 (0.96–1.06) | 0.643 |

**Supplementary Table 9. Sensitivity analysis for incident outcomes excluding events within the first year after MRI.** Sensitivity analysis excluding incident events occurring within the first year after MRI to reduce potential reverse-causation bias. Participants whose event occurred within one year of the MRI scan, and those with less than one year of follow-up, were excluded from each risk set. Fatty pancreas was categorised according to MRI-derived pancreatic PDFF thresholds as <6%, 6 to <16%, and ≥16%. Incident outcomes were defined as the first occurrence more than one year after MRI and were analysed using Cox proportional hazards models, reported as hazard ratios (HRs) with 95% confidence intervals (CIs). Participants were followed from MRI to first event, death, or censoring on March 31, 2023. All analyses were conducted in the complete-case analytic cohort. Models were adjusted for age at imaging, sex, obesity defined as BMI ≥30 kg/m², and high MRI-derived visceral adipose tissue defined as VAT ≥ the cohort-specific 90th percentile; models for CKD were additionally adjusted for type 2 diabetes and hypertension, and models for MACE were additionally adjusted for type 2 diabetes, hypertension, dyslipidaemia, and smoking status. Continuous analyses were performed per 5% increment in pancreatic PDFF. P for trend was calculated by modelling pancreatic fat categories as an ordinal variable. Abbreviations: BMI, body mass index; CI, confidence interval; CKD, chronic kidney disease; HR, hazard ratio; MACE, major adverse cardiovascular events; MRI, magnetic resonance imaging; PDFF, proton density fat fraction; T2D, type 2 diabetes; VAT, visceral adipose tissue.

| **Outcome** | **Pancreatic PDFF** | **Events, n (%)** | **Competing deaths, n (%)** | **Adjusted subdistribution HR (95% CI)** | **p** |
| --- | --- | --- | --- | --- | --- |
| Incident CKD | <6% | 58 (0.9%) | 77 (1.2%) | Ref. | — |
|  | 6 to <16% | 150 (1.7%) | 128 (1.5%) | 1.28 (0.94–1.76) | 0.123 |
|  | ≥16% | 130 (3.6%) | 85 (2.3%) | 1.83 (1.30–2.57) | <0.001 |
|  | P for trend | — | — | — | <0.001 |
|  | Per 5% increase in PDFF | — | — | 1.11 (1.05–1.17) | <0.001 |
| Incident non-fatal MACE | <6% | 140 (2.2%) | 60 (0.9%) | Ref. | — |
|  | 6 to <16% | 340 (4.1%) | 95 (1.2%) | 1.32 (1.07–1.63) | 0.009 |
|  | ≥16% | 178 (5.4%) | 59 (1.8%) | 1.31 (1.02–1.68) | 0.036 |
|  | P for trend | — | — | — | 0.039 |
|  | Per 5% increase in PDFF | — | — | 1.02 (0.98–1.07) | 0.291 |

**Supplementary Table 10. Competing-risk sensitivity analyses for incident CKD and non-fatal MACE.** Competing-risk analyses were performed using Fine–Gray subdistribution hazard models, treating death without the outcome of interest as a competing event. For MACE, analyses were restricted to non-fatal MACE because cardiovascular death was included in the main MACE composite outcome; the competing event was therefore non-cardiovascular death. Fatty pancreas was categorised according to MRI-derived pancreatic PDFF thresholds as <6%, 6 to <16%, and ≥16%. Incident CKD and incident non-fatal MACE were analysed as first events after MRI and are reported as adjusted subdistribution hazard ratios (HRs) with 95% confidence intervals (CIs). All analyses were conducted in the complete-case analytic cohort. Models were adjusted as in the primary analyses: age at imaging, sex, obesity (BMI ≥30 kg/m²), and high visceral adipose tissue (≥90th percentile); the CKD model was additionally adjusted for type 2 diabetes and hypertension, and the non-fatal MACE model additionally for type 2 diabetes, hypertension, dyslipidaemia, and smoking status. Continuous analyses were performed per 5% increment in pancreatic PDFF. P for trend was calculated by modelling pancreatic fat categories as an ordinal variable. Abbreviations: BMI, body mass index; CI, confidence interval; CKD, chronic kidney disease; HR, hazard ratio; MACE, major adverse cardiovascular events; MRI, magnetic resonance imaging; PDFF, proton density fat fraction; T2D, type 2 diabetes; VAT, visceral adipose tissue.
